# Ultra-sensitive pH responsive hydrogels with injectable and self-healing performance for controlled drug delivery

**DOI:** 10.1016/j.ijpx.2025.100334

**Published:** 2025-04-15

**Authors:** Yang Yu, Yili Zhao, Yujiao Zou, Chanyi Lu, Ni Li, Zhiyuan Shi, Xin Li, Xixi Lai

**Affiliations:** a Department of Respiratory and Critical Care Medicine, The First Affiliated Hospital of Wenzhou Medical University, Wenzhou 325015, China; b State Key Laboratory of Bio-based Fiber Materials, College of Textile Science and Engineering, Zhejiang Sci-Tech University, Hangzhou 310018, China; c Tianjin Key Laboratory of Drug Delivery & High-Efficiency, School of Pharmaceutical Science and Technology, Tianjin University, Tianjin 300072, China; d Department of Mechanical and Automation Engineering, The Chinese University of Hong Kong, Hong Kong 999077, China

**Keywords:** Hydrogel, Schiff-base, Ultra pH-sensitive drug release, Injectable, Self-healing

## Abstract

Ultra-sensitive pH-responsive drug delivery system designed to operate within the slightly acidic microenvironment of tumors are highly desired for hydrogel applications in cancer therapy. In this study, 4-Formylbenzoic acid modified polyvinyl alcohol (PVA-FBA, PF) was synthesized and utilized as a carrier for encapsulating the anticancer drug Doxorubicin (Dox). This was subsequently crosslinked with polyethylenimine (PEI) via benzoic-imine bond to form drug-loaded PVA-FBA/PEI hydrogel (D-PFP). The D-PFP hydrogel was characterized using various techniques. The results indicated that the optimal conditions for hydrogel preparation involved using PF-0.25 polymer, which had an aldehyde group content of 0.82 mmol/g, as the precursor, along with a 12 wt% precursor solution for crosslinking with a 5 wt% PEI solution. The resulting hydrogel exhibited good structural stability and favorable morphology. Drug release studies indicated that the hydrogel demonstrated minimal drug leakage under physiological conditions (pH 7.4), while exhibiting a significantly higher drug release rate at pH 6.8, thereby underscoring its superior pH sensitivity. Rheological evaluations further confirmed its injectability and self-healing properties. Moreover, the hydrogel displayed excellent cytocompatibility and significantly inhibited cancer cell activity at pH 6.8. These characteristics suggest the potential of this hydrogel as a drug delivery system with ultra-sensitive drug release properties, particularly for future applications in chemotherapy for cancer.

## Graphical abstract

**Figure:**
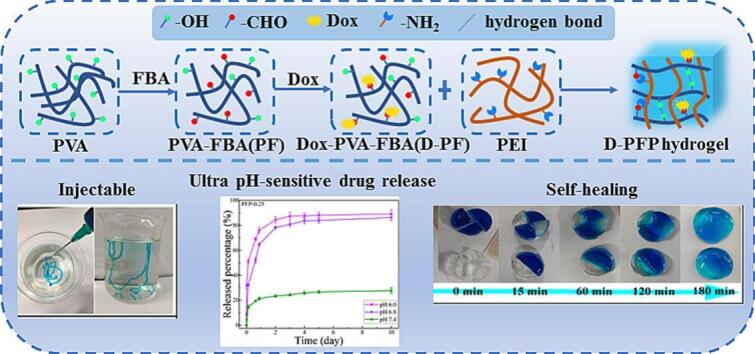

## 1 Introduction

Cancer continues to pose a significant global health challenge, with chemotherapy serving as a fundamental and critical treatment modality (Wei et al., 2021; Wu et al., 2022). By leveraging cytotoxic agents, chemotherapy has played a pivotal role in not only inhibiting tumor growth but also extending the survival of countless patients (Siegel et al., 2021). However, a major obstacle that limits the full potential of chemotherapy is the lack of precise drug release (Lai et al., 2025; Li et al., 2023c; Li et al., 2024b).

In this context, drug delivery systems have emerged as transformative solutions, offering distinct advantages. These systems can encapsulate drugs, thereby protecting them from premature degradation and enabling controlled release over time (Cheng et al., 2023; Kashkooli et al., 2020). Furthermore, they can be engineered to target specific tissues or cells, thereby increasing the local drug concentration at the disease site (Li et al., 2023a; Xin et al., 2016).

Hydrogels present a promising option due to their straightforward synthesis, notable porosity, and exceptional capacity for drug encapsulation (Li et al., 2025). Particularly, injectable hydrogels enable easy administration, allowing for local drug delivery to the targeted site. This not only enhances the therapeutic efficacy by maintaining high drug concentrations where needed but also reduces the potential for systemic side-effects, making injectable hydrogels a highly versatile platform for advanced drug delivery systems (Song et al., 2025; Xiao et al., 2021). By tailoring the composition and structure of hydrogels, it is possible to create drug carriers that respond to specific triggers, such as endogenous or exogenous stimuli (Dreiss, 2020; Sun et al., 2020; Tian and Liu, 2023). The elevated metabolic activity in cancerous tissues results in lactic acid accumulation, shifting the pH from physiological levels (pH 7.4) to mildly acidic conditions (pH 6.5-7.0) (Jayachandran et al., 2023; Kousar et al., 2023; Ranote et al., 2022; Xu et al., 2024). Consequently, pH-responsive hydrogels have garnered considerable attention in the quest for more effective drug delivery solutions, particularly for targeting the mildly acidic microenvironments characteristic of tumors (Chu et al., 2022).

Among various options, polyvinyl alcohol (PVA)-based hydrogels have emerged as a favored choice due to their biocompatibility and versatility. Multiple acid-responsive bonds can be incorporated into PVA hydrogels to impart pH sensitivity (Alipournazari et al., 2024; Rahmani et al., 2023; Suhail et al., 2023). For instance, Geng et al. utilized Schiff base bonds to construct acid-responsive gelatin/PVA hydrogels (Geng et al., 2021). The reversible nature of Schiff base formation and protonation under varying pH conditions enables the hydrogel to respond effectively to acidic stimuli. Moreover, PVA-based hydrogels have demonstrated excellent self-healing and injectable properties. Li et al. found that the multiple dynamic reversible bonds within collagen peptide-based hydrogels facilitated spontaneous repair of mechanical damage (Li et al., 2024a). This self-healing capability is crucial for maintaining the integrity of the hydrogel in vivo, thereby ensuring continuous drug release functionality. Shan et al. demonstrated that PVA hydrogels could be easily extruded through a syringe needle, providing a convenient and efficient method for in situ drug delivery (Shan et al., 2024). However, the relatively high drug release at physiological pH poses challenges for their application, leading to increased drug loss and limited precision in managing narrow pH fluctuations.

Among various pH-sensitive dynamic covalent bonds, such as imines, acylhydrazones, and borates, imine bonds, which are formed through the condensation of aldehydes or ketones with amines (Schiff bases), represent an appealing choice (Tidwell, 2008). The stability of imine bonds is influenced by the chemical environment, particularly the concentration of hydrogen ions. Modifying the chemical structure of the substrate offers a means to regulate this stability. Crugeiras et al. investigated the formation of Schiff bases from benzaldehyde with various substituents, demonstrating their impact on the equilibrium constants of the resulting benzoic-imines (Crugeiras et al., 2009). Research indicates that imine bond stability increases at physiological pH due to π-π stacking interactions between the carbonyl group and the benzene ring. Consequently, benzoic-imine bonds serve as ideal substrates for the construction of pH-responsive hydrogels and their application in controlled drug release. Building on this understanding, developing smart hydrogels with excellent physiological stability, ultra-sensitive acid responsiveness, injectability, and self-healing properties based on Schiff base bonds containing benzene rings holds great promise for controlled drug delivery.

**Fig. 1:**
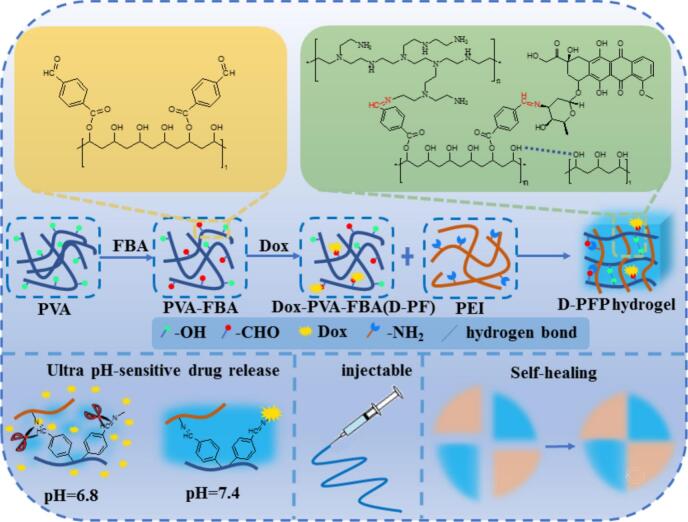
Reaction scheme for the preparation of D-PFP hydrogel. PVA is initially reacted with FBA through esterification to yield PVA-FBA. Subsequently, Dox is conjugated to PVA-FBA via a Schiff base reaction. The resulting mixture is then combined with PEI to form the D-PFP hydrogel. The prepared hydrogel exhibits ultra-sensitive drug release behavior at pH 6.8, while also demonstrating excellent injectability and self-healing properties.

In this study, 4-Formylbenzoic acid modified polyvinyl alcohol (PVA-FBA, PF) was synthesized and utilized as a carrier for encapsulating the anticancer drug Doxorubicin (Dox), and subsequently crosslinked with polyethylenimine (PEI) via benzoic-imine bonds to form a drug-loaded PVA-FBA/PEI hydrogel (D-PFP). The optimal parameters for hydrogel preparation involved using PF-0.25 polymer, which contained 0.82 mmol/g of aldehyde groups, as the precursor, along with a 12 wt% precursor solution for crosslinking with a 5 wt% PEI solution. The prepared hydrogels exhibited excellent stability, ultra-sensitive pH responsiveness, as well as injectable and self-healing properties. This approach provides a novel strategy for designing advanced drug delivery systems with ultra-sensitive drug release characteristics and holds significant potential for future applications in chemotherapy for cancer (Fig. 1).

## 2 Materials and methods

### 2.1 Materials

Polyvinyl alcohol (PVA, Mw = 67,000, Hydrolysis: 88 %), 4-dimethyl-aminopyridine (DMAP, 99 %), Na_2_SO_4_, Dimethyl sulfoxide (DMSO, 95 %), Phosphate buffer (PBS; NaCl, KCl, Na_2_HPO_4_, KH_2_PO_4_), Methyl blue (MB) and 4-Formylbenzoic acid (FBA, 98 %) were purchased from Aladdin (Shanghai, China). 1-(3-Dimethylaminopropyl)-3-ethylcarbodiimide hydrochloride (EDC, 99 %) was purchased from J&K Chemical Ltd. (Beijing, China), Polyethylenimine (PEI, Mw = 25,000) was obtained from Sigma-Aldrich (St Louis, MO). Doxorubicin hydrochloride (Dox.HCl, 99 %) was purchased from HvsF United (Beijing, China). Water used in all experiments was purified using a Milli-Q Plus 185 water purification system (Millipore, Bedford, MA) with a resistivity higher than 18.2 MΩ·cm.

### 2.2 Synthesis of PVA-FBA

The benzaldehyde-modified PVA was synthesized via an esterification reaction between PVA and FBA. Initially, PVA (1 g) was dissolved in DMSO (6 mL) in a water bath at 60 °C for 5 h. Once the solution became clear, anhydrous Na_2_SO_4_ (0.01 g) was added to facilitate the removal of water. Subsequently, the carboxyl group of FBA (0.5 g) was activated using EDC (0.65 g) and DMAP (0.065 g) dissolved in DMSO (3 mL), stirring for 20 min. DMSO was selected as the solvent due to its ability to effectively dissolve both PVA and FBA, while also suppressing the hydrolysis of EDC/DMAP and the DMAP-ester. Meanwhile, the PVA solution was cooled in an ice bath and agitated. The acti*v*ated FBA solution was then added incrementally to the PVA solution at five different weight ratios (0.1, 0.2, 0.25, 0.33, and 0.5 for FBA: PVA). The addition of the FBA solution resulted in a rapid yellow color change in the mixture. The reaction was allowed to proceed at room temperature for 24 h. Following this, the solution was dialyzed for 4 days using a membrane with a molecular weight cut-off of 8000–14,000 Da. This dialysis process involved one day in PBS and three days in deionized water to remove DMSO and unbound molecules. After the completion of dialysis and subsequent drying, the resulting yellowish product was identified as PVA-FBA (PF). The polymers synthesized using the five different feed ratios were designated as PF-0.1, PF-0.2, PF-0.25, PF-0.33, and PF-0.5, respectively.

### 2.3 Preparation of PFP hydrogels

The hydrogels were prepared by mixing solutions of PF (10 mL) and PEI (200 μL, 5 %) dissolved in deionized water. Upon combining these solutions in specific proportions (with PF concentrations of 6 %, 9 %, 12 %, 15 %, and 18 % *w*/*v*) and stirring at room temperature, the PVA-FBA/PEI (PFP) hydrogel formed immediately.

For the synthesis of D-PFP hydrogels, a predetermined quantity of Dox (2 mg) was added to the PF solution (1 g) for pre-binding. The resulting mixture was agitated at 37 °C for 10 min, promoting incorporation of Dox into the PF polymer through a Schiff base reaction, ultimately yielding the Dox-loaded PVA-FBA polymer (D-PF). Subsequently, following the established methodology for preparing the PFP hydrogel, the PF polymer in the hydrogel formulation was replaced with the D-PF polymer, resulting in the formation of the D-PFP hydrogel.

### 2.4 Characterizations

The ^1^H NMR spectrum of the product was obtained using a Bruker Avance AV400 MHz spectrometer (Bruker, Germany). For sample preparation, 10 mg of PVA and PVA-FBA samples were dissolved in DMSO‑*d*_6_ by heating, resulting in transparent solutions suitable for analysis. The hydrophilic properties of various PVA-FBA samples (PF-0.1, PF-0.2, PF-0.25, PF-0.33, and PF-0.5) were evaluated by measuring the water contact angle using an Optical Contact Angle measurement system (OCA JCY-2, Data Physics Instruments GmbH, Germany). For contact angle measurements, a 5 μL droplet of deionized water was gently dispensed onto each sample, and the static contact angle was determined by averaging measurements from at least five observations. The contact angle measurements adhered to a three-point measurement methodology.

Fourier transform infrared (FTIR) spectroscopy was performed using a Thermo Nicolet FTIR (NICOLET Is50) to analyze the functional groups and chemical bonds present in the PF polymer and PFP hydrogel. FTIR spectra were acquired in the wavenumber range of 500–4000 cm^−1^ with a spectral resolution of 4 cm^−1^ using an Attenuated Total Reflection (ATR) configuration. The porous structure of different PFP hydrogels (PFP-0.2, PFP-0.25, and PFP-0.33) was examined using scanning electron microscopy (JSM-5610LV, JEOL, Japan). For each sample, pore size was analyzed using ImageJ 1.40G software. All hydrogel specimens were lyophilized and subsequently freeze-fractured in liquid nitrogen for analysis.

### 2.5 Aldehyde quantification via titration

The aldehyde content in the PF was quantified using hydroxylamine hydrochloride titration. This method is based on the quantitative reaction between hydroxylamine hydrochloride and aldehyde, leading to the formation of oxime and the simultaneous release of hydrochloric acid (HCl) (Lou et al., 2020; Paiva et al., 2016). The concentration of aldehyde groups was subsequently calculated by titrating the resulting hydrochloric acid with a standard sodium hydroxide (NaOH). To perform the titration, 0.10 g of PF was dissolved in 5 mL of deionized water. A precisely measured 20.00 mL of 0.25 mol/L hydroxylamine hydrochloride solution, adjusted to a pH of 5.0, was then added to the PF solution. After a 4-hour reaction at 40 °C, the solution was titrated back to pH 5.0 using a 0.05 mol/L NaOH solution. Additionally, a blank titration was conducted using 0.10 g of dried PVA. The aldehyde content of PF (mmol/g) was calculated using Eq. (1):

(1) $$\text{Aldehyde content}\left(\text{mmol}/g\right)=\frac{\left(V_{1}-V_{2}\right)\times c}{m}\times 100$$

where V_1_ and V_2_ are the volumes of NaOH consumed by PVA-FBA and the original PVA during titration (mL). c is the molar concentration of NaOH solution (mol/L). m is the weight of PF polymer (g).

### 2.6 Gel fraction analysis

Sol-gel analysis was conducted on unwashed hydrogel samples to determine the fraction of crosslinked polymer within their structure. Initially, the hydrogel samples were sectioned into specific dimensions, and their wet weight was recorded. These hydrogel disks were then immersed in 250 mL of water for 4 h. Following this period, the swollen hydrogel disks were dried at 40 °C until complete desiccation was achieved (W_d_), using Na_2_SO_4_ as a desiccant. Concurrently, samples that were not subjected to soaking were measured after drying, these are referred to as the original dry weight (W_d0_). Multiple groups were tested in parallel. The gel fraction was subsequently determined using Eq. (2):

(2) $$\mathrm{Gel}\;\mathrm{fraction}\left(\%\right)=\frac{W_{d}}{W_{d0}}\times 100$$

where W_d_ represents the dried weight of the hydrogel sample after soaking for a specified duration, and W_d0_ indicates the dried weight of the original hydrogel sample. All data were normalized based on wet weight measurements.

### 2.7 Swelling studies and stability assessment

The swelling properties of the hydrogel were assessed using gravimetric analysis (Peng et al., 2023). Each lyophilized PFP hydrogel specimen (W_L_) was individually immersed in deionized water and PBS (pH 7.4) at room temperature. At defined intervals, the swollen gel was removed, excess liquid was blotted away using absorbent paper, and the weight (W_t_) was recorded multiple times, while the process was repeated in parallel for multiple groups. The swelling ratio (SR) was calculated using Eq. (3):

(3) $$\mathrm{SR}\left(\%\right)=\frac{W_{t}-W_{L}}{W_{L}}\times 100$$

where W_L_ and W_t_ denote the mass of the hydrogel specimens before immersion and after a specific swelling duration, respectively.

Gel stability tests were conducted using a similar methodology to evaluate the degree of degradation. Multiple sets of parallel samples were prepared and collected at various time intervals over the course of a month for drying and weighing. Instead of lyophilization, a low-temperature drying method was employed to obtain the dry weight of the samples. This approach mitigated issues related to sample fragmentation and adhesion to petri dishes, ensuring accurate measurements of hydrogel degradation. The degree of degradation of the hydrogel (Hd) can be expressed using Eq. (4):

(4) $$\mathrm{Hydrogel degradation}\;\left(\%\right)=\left(1-\frac{W_{\mathrm{dt}}}{W_{d0}}\right)\times 100$$

where W_dt_ represents the dried weight of the hydrogel sample after soaking for time t, and W_d0_ indicates the dried weight of the original hydrogel sample prior to soaking. All data were normalized based on wet weight measurements.

### 2.8 In vitro drug encapsulation and release of D-PFP hydrogel

To evaluate drug encapsulation in PFP hydrogels, samples (PFP-0.2, PFP-0.25, and PFP-0.33, 12 wt%) containing 2 mg/g of Dox were prepared following the afore-mentioned procedure. One gram of D-PFP hydrogel was placed in a vial, and subsequently, 15 mL of PBS (pH 7.4) was added. The hydrogel was then incubated in a 37 °C shaker at a shaking frequency of 30 rpm. After a 3-hour immersion period, 2.5 mL of solution was withdrawn from each vial and subjected to UV spectroscopic analysis to assess drug release. The drug encapsulation efficiency in D-PFP hydrogels was evaluated through three parallel trials using Eq. (5):

(5) $$\mathrm{Encapsulation efficiency}\;\left(\%\right)=\left[\frac{M_{T}-M_{3}}{M_{T}}\right]\times 100\%$$

where M_3_ represents the mass of Dox released after 3 h, and M_T_ indicates the initial total quantity of drug added to the sample. The concentration of Dox was determined using the Beer-Lambert Law and a Dox standard curve measured with a Lambda 950 UV–visible spectrophotometer.

The drug release performance of PFP-0.25 hydrogels in different environments was assessed using the same methodology, with the exception that the hydrogels were immersed in various PBS solutions (pH 6.8, and 7.4) over 10 days. At designated intervals, 2.5 mL of solution was withdrawn from each sample vial, and fresh PBS was added to maintain the total volume. Drug release was monitored by analyzing the sample solutions using UV spectroscopy, with three parallel experiments conducted. The cumulative release mass (M_t_) and release ratio (R_r_) of Dox from the D-PFP hydrogel at different time points were calculated using Eqs. (6) and (7): (Liu et al., 2023).

(6) $$M_{t}=C_{t}\times V_{a}+\sum \left(C_{t-1}\times V_{r}\right)$$

(7) $$R_{r}=\left(M_{t}/M_{T}\right)\times 100\%$$

where M_t_ denotes for the cumulative release mass of Dox at time t, and M_T_ represents the initial total quantity of drug added to the sample. The concentration of Dox at time t (C_t_) was determined using the Beer-Lambert Law and a measured Dox standard curve. The overall volume of PBS in the vials is denoted as V_a_, and V_r_ indicates the volume withdrawn from the vials during each sampling instance (V_r_ = 2.5 mL). R_r_ is defined as the release ratio of Dox released from the samples at time t.

To investigate the correlation between the hydrogel's drug release and degradation, an influence factor was calculated by dividing the measured drug release rate by the degree of degradation, both assessed post-incubation using Eq. (8):

(8) $$\mathrm{Influence factor}=\frac{R_{r}}{H_{d}}$$

where R_r_ is the release ratio of Dox released from the samples at time t, and H_d_ denotes the hydrogel degradation degree.

### 2.9 Rheology measurement

The MCR-52 Rotary Rheometer (Anton Paar, Austria) was employed to evaluate various rheological parameters at room temperature. A consistent gap of 0.5 mm between the plate and the hydrogel samples was maintained throughout the experiments. The hydrogels were incubated for 1 h prior to testing. Dynamic frequency sweep experiments were conducted by applying frequencies ranging from 1 to 100 Hz at a 100% amplitude. Subsequently, variable strain amplitudes from 0% to 1000 % were applied at 1 Hz, and the modulus values were recorded to assess the mechanical properties of the hydrogel under strain. Additionally, a visual compression experiment was performed on the PFP hydrogel. The cylindrical PFP hydrogel was subjected to different loading pressures (e.g., finger pressure, 500 g, and 1000 g) for a duration of 30 min, after which the force was removed to observe changes in the shape of the PFP hydrogel.

The self-healing capability of the hydrogel was also assessed. Initially, an alternating step strain was applied, transitioning from γ = 1 % to γ = 700 % at a fixed angular frequency of 1 Hz, with each strain interval lasting 60 s. Subsequently, PFP-0.25 hydrogels, one dyed with methyl blue and the other untreated, were sectioned into three segments. These segments were recombined and stored at 37 °C for 3 h to facilitate the healing process. Several samples were prepared for further analysis, with the healing duration varying; the cut PFP-0.25 hydrogels were allowed to heal at 37 °C for 1, 2, and 3 h, respectively. The storage modulus of both the initial and healed hydrogels was measured. The healing efficiency (HE) was calculated using Eq. (9):

(9) $$\mathrm{HE}\left(\%\right)=\frac{S_{H}}{S_{O}}\times 100$$

where S_H_ represents the storage modulus of the healed hydrogel and S_O_ denotes the storage modulus of the original hydrogel.

### 2.10 Cytocompatibility evaluation

PFP hydrogels with varying concentrations (0, 10, 25 and 50 mg/mL) were aseptically immersed in PBS under pH 7.4 and pH 6.8 at 37 °C for 48 h to generate leach solutions with differing concentrations. Mouse fibroblast (L929) cells were seeded at a density of 10^4^ cells/well in a 96-well plate containing 100 μL of 1640 medium supplemented with 10 % fetal bovine serum (FBS) and 1 % antibiotic solution. After a 24-hour incubation period, the medium was aspirated. Subsequently, 10 μL of the PFP hydrogel leach solutions were diluted with growth medium to achieve final concentrations of 0, 1, 2.5 and 5 mg/mL. This diluted solution was added to six replicates of the previously incubated cells and further incubated at 37 °C for an additional 24 h. Cytocompatibility was assessed using the CCK-8 assay method.

### 2.11 In vitro anti-cancer cells study

The D-PFP hydrogel underwent a 48-hour leaching process under varying pH conditions, and the resulting leachate was utilized to interact with rat glioma (C6) cells. The objective was to assess the drug release behavior from the D-PFP hydrogel and its impact on suppressing cancer cells under different pH within a specific application context. D-PFP hydrogels (PFP-0.25, 12 wt%) containing Dox were synthesized following the afore-mentioned procedure. In the experimental group, different samples were leached in PBS to achieve different concentrations of the anticancer drug Dox (0, 2, 8, and 32 μg/mL). The experimental groups were organized to maintain consistency in the Dox content across samples within each group, adhering to a standardized approach.

The experimental setup involved introducing drug-free PFP hydrogel and D-PFP hydrogel into 5 mL of PBS solution at pH 7.4 and pH 6.8. Additionally, a control group was established by introducing equal amounts of Dox into 5 mL of PBS solution at pH 7.4 and a standard PBS solution (5 mL, pH 7.4) utilized as a control for comparative purposes. C6 cells were initially seeded at a density of 10^4^ cells/well in a 96-well plate containing 100 μL of H-DMEM medium. After allowing 24 h for cell adhesion, the growth medium was removed. Subsequently, 10 μL of the PFP hydrogel and D-PFP hydrogel leach solutions were diluted to a final volume of 100 μL using growth medium. This diluted extract was then added to six replicates of the pre-incubated cells in the 96-well plate and incubated at 37 °C for an additional 24 h. In vitro anticancer activity was assessed using the CCK-8 assay method.

Furthermore, a calcein-AM/PI assay was conducted to evaluate the anticancer activity of the hydrogel. Briefly, 4 T1 cells at a concentration of 10^4^/well were seeded in a 48-well plate and cultured for 24 h, after which the cells were treated with PBS, PFP hydrogel, or D-PFP hydrogel. The medium was subsequently discarded, and the cells were washed three times with PBS. The cells were then co-stained with calcein-AM/PI. After 20 min, images were captured using an inverted fluorescence microscope (Olympus FV1200 confocal microscope).

## 3 Results and discussion

### 3.1 Synthesis and characterization of PF polymers and PFP hydrogels

**Fig. 2:**
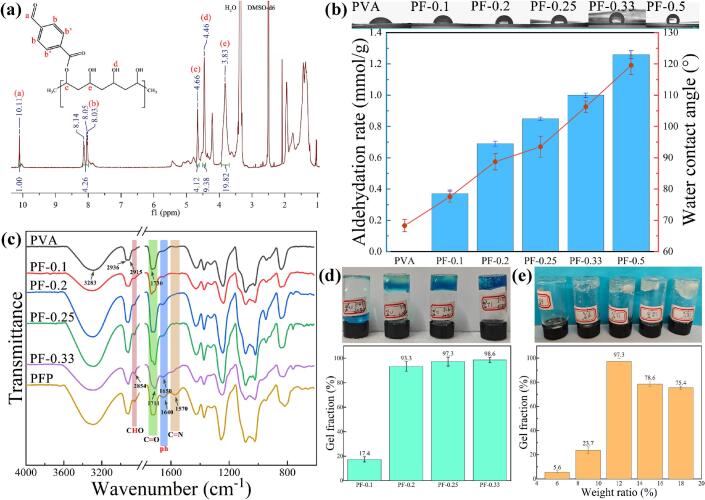
(a) ^1^H NMR spectrum of benzaldehyde-modified PVA; (b) Aldehyde content and hydrophilic properties of samples with varying feed ratio; (c) FTIR spectra of PVA, PF samples with varying feed ratios, and PFP hydrogels; (d) Gel fraction in PFP hydrogels corresponding to PF materials with differing aldehyde content and (e) Gel fraction in hydrogel precursors with varying concentrations.

The PVA-FBA was verified through ^1^H NMR spectroscopy (Fig. 2a). The signals observed at 10.11 ppm and 8.03–8.14 ppm correspond to the aldehyde group and the protons of the benzene ring in PVA-FBA, respectively. Additionally, the signal at 4.66 ppm represents the methyne proton on the PVA chain that is linked to the ester bond (Guerrero et al., 2019). The characteristic peaks at 4.46 ppm and 3.83 ppm correspond to the hydroxyl and methylene protons of PVA, respectively (as compared in Fig. S1) (Li et al., 2023b). The modification content of FBA in PVA was determined through hydroxylamine hydrochloride titration. An increase in the FBA ratio (0.1–0.5) resulted in a corresponding increase in the aldehyde group content in PVA-FBA, ranging from 0.37 mmol/g to 1.26 mmol/g, although the yield decreased from 56 % to 37 % (Table 1). This reduced yield suggests limitations in the facile modification of functional groups on the PVA chain, along with increased steric hindrance due to the benzene ring structure of FBA, both of which inhibit further esterification.

**Table 1:** The Aldehyde content and yield percentage of PF polymers synthesized with varying feed ratios.

Sample codes	Feed ratio PVA/FBA	Aldehyde content^a^ (mmol/g)	Yield percentage^b^ (%)
PVA	1:0	0	N/A
PF-0.1	1:0.1	0.37	56
PF-0.2	1:0.2	0.69	56
PF-0.25	1:0.25	0.85	51
PF-0.33	1:0.33	1.09	48
PF-0.5	1:0.5	1.26	37

a The aldehyde content of PF was accomplished by hydroxylamine hydrochloride titration.

b Yield percentage represents the ratio of actual production to theoretical production.

Similarly, the increased content of aromatic rings elevates the water contact angle of the material from 68.3° to 119.5° (Fig. 2b), changing its nature from hydrophilic to hydrophobic. PF-0.5 exhibits low water solubility and requires ethanol for dissolution. Therefore, the complexities involved in preparing a PF-0.5 solution render it unsuitable as a precursor for hydrogel formation. The Schiff base structure of the hydrogel imparts reversible pH-responsive behavior. Upon the addition of an acid, the gel transitions into a sol state, while it regains its gel form upon the addition of a base (Fig. S2).

To confirm the structure of PVA-FBA and assess the chemical changes that occur upon reaction with PEI, FTIR analysis was conducted (Fig. 2c). For the PVA sample, the observed absorption peaks at 3283 cm^−1^, 2936 cm^−1^, and 2915 cm^−1^ are attributed to the stretching vibrations of hydroxyl (—OH) and methylene (—CH₂—) groups, respectively. The absorption peak at 1730 cm^−1^ corresponds to the stretching vibration of the ester carbonyl group. These peaks indicate the presence of residual acetate groups from the incomplete alcoholysis of PVA during its production. In the case of the PVA-FBA sample, the peak at 2854 cm^−1^ is attributed to the C—H stretching vibration of the aldehyde group. The distinctive stretching vibration observed at 1711 cm^−1^ corresponds to a newly introduced carbonyl group (C=O, aldehyde), which is distinct from the stretching vibration exhibited by the carbonyl group (C=O, ester) at 1730 cm^−1^ (Abdelghafour et al., 2022; Takacs et al., 2022). Furthermore, the peak at 1650 cm^−1^ is due to the C=C stretching in the aromatic ring. The formation of an intramolecular hydrogen bond between the CHO and OH groups of PF results in an increase in intensity and a shift in the wa*v*enumber of the unmodified OH group of the PVA backbone. This discrepancy serves to confirm the occurrence of the modification reaction.

Following the successful preparation of PVA-FBA, PEI was introduced to the PVA-FBA solution to synthesize the hydrogels. As shown in Fig. 2c, the PFP hydrogel exhibits a distinct broad peak at 1640 cm^−1^, which overlaps with the pre-existing peak associated with the benzaldehyde group. Additionally, the presence of a new peak at 1570 cm^−1^ confirms the reaction between the NH₂ group of PEI and the CHO group of PVA-FBA, solidifying the chemical structure of the hydrogel. This observation serves as evidence for the formation of Schiff bases (—CH=N—) (Zhao et al., 2017).

### 3.2 Gelling property

The gelling properties of PFP hydrogels were analyzed by determining the gel fraction. Notably, the PFP-0.1 gel exhibits only a 17.4 % gel fraction, indicating its low aldehyde content, which is insufficient to generate a stable three-dimensional hydrogel network via imine bond formation. As the functional group content increases, the stability of the hydrogel significantly improves, with gel fractions ranging from 93.3 % to 98.6 % for PFP-0.2, PFP-0.25, and PFP-0.33 (Fig. 2d). Various concentrations of the PF-0.25 solution (6 %, 9 %, 12 %, 15 %, and 18 % *w*/*v*) were employed for hydrogel preparation by mixing separate solutions of PF and PEI (*w*/w, 5 %). The optimal concentration for hydrogel formation was determined to be approximately 12 %, while concentrations below 9 % were inadequate for stable gel formation. Conversely, concentrations exceeding 15 % resulted in poor dissolution and rapid gelation, leading to insufficient gel crosslinking, as visually observed (Fig. 2e). Consequently, for subsequent experimental investigations, three samples (PF-0.2, PF-0.25, and PF-0.33) were selected to prepare a solution with a mass fraction of 12 %.

### 3.3 Swelling and stability properties of hydrogels

**Fig. 3:**
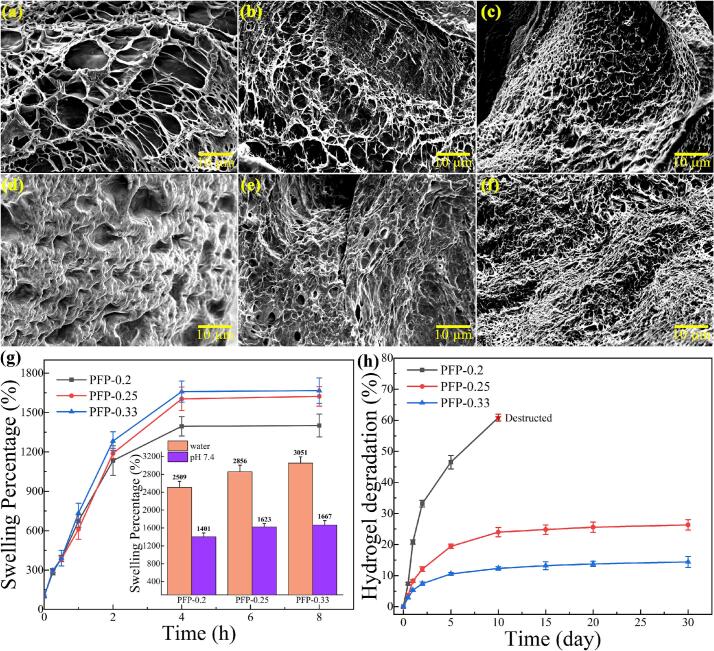
Cross-sectional SEM images of PFP hydrogels before (PFP-0.2, a; PFP-0.25, b; PFP-0.33, c) and after 4 h of immersion in PBS (PFP-0.2, d; PFP-0.25, e; PFP-0.33, f); (g) Swelling properties and (h) stability tests of PFP hydrogels.

SEM images clearly indicate a reduction in pore size within the hydrogel as the FBA modification ratio of the PF polymer increases (Fig. 3a–c). The calculated pore sizes for the PFP-0.2, PFP-0.25, and PFP-0.33 specimens were 4.35, 1.98, and 1.31 μm, respectively (Fig. S3). This reduction may be attributed to the increased concentration of aldehyde groups, which facilitates the formation of benzoic-imine bonds, thereby allowing for the establishment of a denser and more compact three-dimensional polymer network. Subsequently, the PFP-0.2, PFP-0.25, and PFP-0.33 specimens were submerged in phosphate-buffered saline (PBS) solution for 4 h, followed by freeze-drying to obtain SEM images (Fig. 3d–f). The images reveal discernible surface reconfiguration and increased roughness. While the overall pore structure of the PFP-0.25 and PFP-0.33 samples remains unchanged after immersion, the PFP-0.2 sample experiences pore healing and eventual disappearance. The stability of hydrogels in aqueous environments necessitates achieving sufficient density in their polymer network.

The swelling properties of freeze-dried PFP hydrogel samples were evaluated by immersion in deionized water and PBS, respectively. The swelling process was typically completed within approximately 6 h (Fig. 3g). The swelling rate of the hydrogels exhibited an increasing trend corresponding to the rise in aldehyde content, ranging from 1401 % to 1667 %. This increase in cross-linking density concurrently enhances the hydrogels' capacity for swelling. Notably, the inset image illustrates that PBS demonstrates a significant inhibitory effect on hydrogel swelling compared to deionized water; this effect may be attributed to the Hofmann effect of ions that restrict the mobility of the polymer macromolecular chains. Although the swelling rates of the three samples do not exhibit significant disparities, it is noteworthy that the PFP-0.2 sample displays a lower final swelling rate. This can be attributed to insufficient aldehyde content for establishing a stable molecular chain network, resulting in hydrogel disintegration in the solution environment.

This phenomenon was further corroborated by the stability test of the hydrogels (Fig. 3h). During the one-month stability assessment, notable differences emerged among the samples. PFP-0.2 exhibited rapid disintegration, losing its stable structure after the tenth day and becoming unmeasurable due to a muddy consistency. In contrast, PFP-0.25 and PFP-0.33 demonstrated degradation rates of 26.34 % and 14.41 %, respectively, signifying a higher degree of stability in comparison. Consequently, the stability test results indicate that the PFP-0.2 hydrogel cannot maintain stability in an aqueous environment and is unsuitable for long-term applications.

### 3.4 In vitro drug encapsulation analysis

**Fig. 4:**
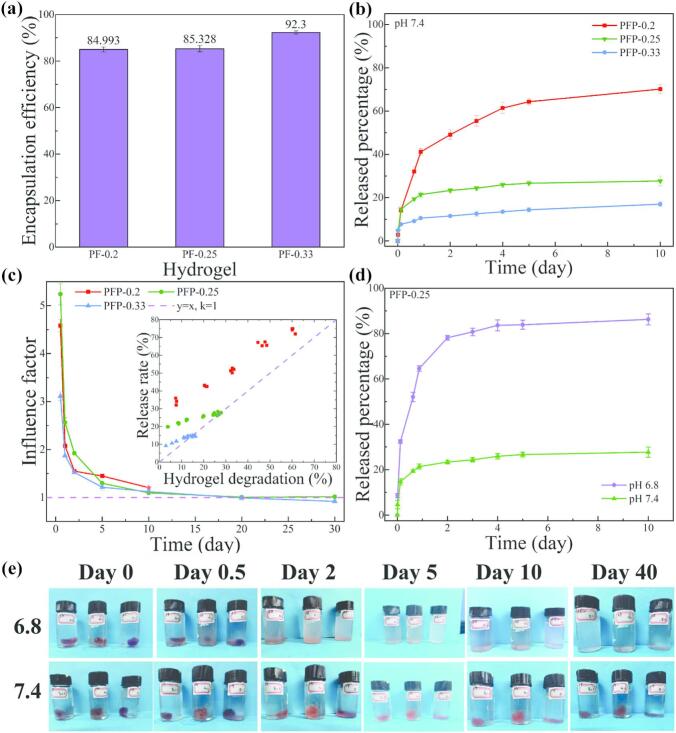
(a) Dox encapsulation rate of the hydrogel; (b) drug release percentage of different PFP hydrogels at pH 7.4; (c) correlation between drug release rates and hydrogel degradation degree; (d) drug release rate of PFP-0.25 at different pH levels; (e) imaging recordings of the experiment.

Encapsulation efficiency refers to the proportion of drug entrapment within the carrier system compared to the total amount of drug initially incorporated into the formulation. This metric serves as a critical evaluation parameter for drug-controlled release systems. In this study, the hydrogel precursor, PF, which contains a significant number of aldehyde functional groups, is capable of binding with the model drug, Dox, thereby reducing the undesired release of the drug (Buffa et al., 2018). The UV–Vis spectra and standard curve for Dox are illustrated in Fig. S4, where Dox exhibits a characteristic absorption peak at 481 nm, demonstrating a strong linear correlation between drug concentration and absorbance. In Fig. 4a, the encapsulation efficiency of the hydrogels increases from 84.99 ± 1.05 % to 92.30 ± 0.65 %. This increase may be attributed to the elevated aldehyde group content, which results in a denser hydrogel structure that facilitates enhanced drug loading. Hydrogels with higher cross-linking densities possess a more compact architectural configuration, thereby reducing the likelihood of drug molecules leaching from the matrix (Sarmah et al., 2023).

Furthermore, the encapsulation of Dox within the hydrogel is influenced by the interaction between PF and Dox, as confirmed by FTIR analysis (Fig. S5a). As previously described, the peaks observed at 2840 cm^−1^ and 1711 cm^−1^ confirm the successful modification of PF with FBA, while the appearance of peaks at 1570 cm^−1^ indicates the formation of —C=N— moieties in PFP. Notable spectral changes occur upon mixing with Dox. In the case of D-PVA, a newly emerged minor peak at 1580 cm^−1^ suggests the presence of free amino groups, indicating unbound Dox within the composite. For D-PF, two additional minor peaks at 1580 cm^−1^ and 1545 cm^−1^ are detected. The peak at 1545 cm^−1^ characterizes the formation of imine bonds resulting from the interaction between Dox and the aldehyde group within PF. Importantly, these imine bonds exhibit a discernible wavenumber shift compared to benzoic-imine bonds.

A composite was developed by combining Dox with PVA, PF-0.25, and PF-0.33 solutions, which were then dehydrated to produce a film. This film was enclosed within a dialysis bag and immersed in deionized water, followed by incubation in PBS at 37 °C on a shaker for 48 h to assess its drug-binding capacity. The drug release test (Fig. S5b) reveals that the PF-0.25 (31.34 %) and PF-0.33 (24.03 %) samples exhibit significantly lower drug release rates compared to the PVA sample (67.12 %). As shown in the imaging recordings (Fig. S5c), the PVA group (left), which lacks benzaldehyde groups, demonstrates a notably higher release of Dox over time in comparison to the PF-0.25 (middle) and PF-0.33 (right) groups. This highlights the significant drug-binding capacity of the PF polymers when combined with Dox.

### 3.5 pH responsive drug release

In pursuit of the envisioned hydrogel application, Dox-loaded PFP hydrogels with varying aldehyde group contents (PFP-0.2, PFP-0.25, and PFP-0.33) were evaluated in a simulated physiological environment at pH 7.4. This evaluation aimed to characterize the long-term drug storage capability of the hydrogels (Fig. 4b). Over 60 % of Dox was released within five days, indicating that PFP-0.2 lacks the stability required for medium- to long-term controlled drug release applications. This finding was further substantiated by photographic evidence (Fig. 4e, in all smaller images depicted, from left to right, PFP-0.33, PFP-0.25, and PFP-0.2, respectively), which shows the loss of structural integrity of PFP-0.2 when immersed in pH 7.4 PBS over time, causing it to flatten at the bottom of the container. In sharp contrast, samples with higher aldehyde group content demonstrate robust long-term drug storage capabilities. As can be clearly seen from Fig. S6, the drug release percentage at the first 50 h was slowly increased. Within the first 50 h of experimentation, 12.5 % of Dox was slightly released from PFP-0.33, while 24.3 % was released from PFP-0.25. This early release can be attributed to the dissolution and diffusion of unbound drug components within the PBS medium, coupled with the progressive disintegration of partially unstable gel structures. However, after 10 days of immersion in PBS (pH 7.4), 83.0 % of the drug in PFP-0.33 and 72.3 % in PFP-0.25 remained steadfastly retained (Fig. 4b). This underscores that the formation of a sufficiently dense Schiff base network within the hydrogel imparts stability and enhances the drug encapsulation capacity, making it suitable for prolonged drug release scenarios.

Fig. 4c illustrates the correlation between drug release rates and the degree of hydrogel degradation from parallel tests. While a consistent pattern at the data point level is absent due to the destructive nature of measuring degradation, a clear relationship between soaking duration and degradation degree emerges. Initially, when the degradation degree is low, drug release exceeds the degradation degree by a factor of 4 for PFP-0.33 and by a factor of 5 for PFP-0.25, indicating the presence of unbound drug and unstable portions of the hydrogel. Over time, this ratio gradually converges toward 1, underscoring the dependence of drug release from the PFP hydrogel on its degradation. The degradation of PFP-0.2 could not be accurately measured after 10 days due to excessive degradation. Notably, PFP-0.33 exhibits an influence factor lower than 1, which is attributed to the interaction between the PF polymer and Dox, as detailed in Fig. S5. Additionally, the high aldehyde content in PFP-0.33 accelerates gel formation, impeding complete precursor intermixing and resulting in uneven drug distribution and an excess of aldehyde groups. This non-uniformity may contribute to a lower drug release rate relative to the rate of hydrogel degradation.

Simultaneously, the influence of varying pH conditions on the drug release rate of D-PFP-0.25 samples was examined (Fig. 4d). In acidic environments, including mildly acidic conditions at pH 6.8, the drug release released slowly during the first 50 h and reach to 86.2 % after 10 days (Fig. S6). This release corresponds visually with a disintegration of the hydrogel's structural integrity (Fig. 4e, first row, middle samples of each photo). In contrast, the hydrogel immersed in a pH 7.4 environment maintains its structural integrity (Fig. 4e, second row, middle samples of each photo) with a markedly lower 10-day release rate of only 27.7 %. This can be attributed to the stability of the benzoic-imide bonds constituting the PFP Schiff hydrogel network at physiological pH, as well as the breakdown that occurs in slightly acidic environments. Thus, these observations confirm the relationship between drug release rate and degradation degree, suggesting that PFP-0.25 and PFP-0.33 hydrogels possess good pH-responsive drug release capabilities.

### 3.6 Rheological characterization of injectable and self-healing properties

**Fig. 5:**
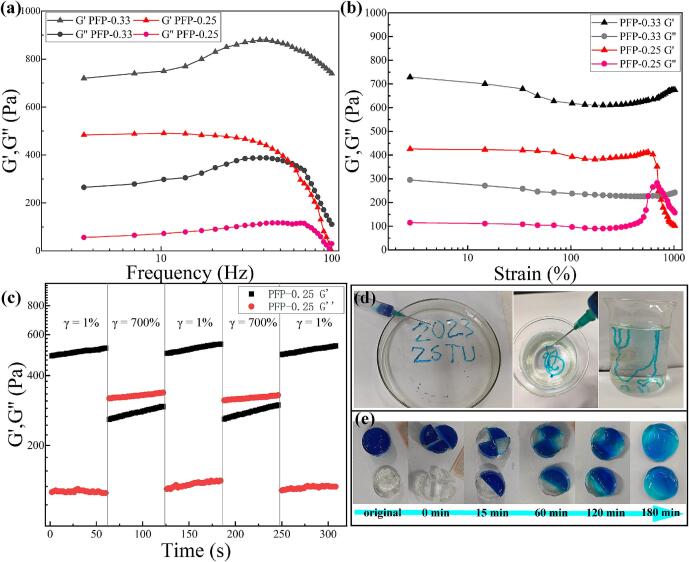
Rheological property testing, (a) frequency scanning, (b) strain scanning of PFP hydrogels; (c) self-healing performance of the hydrogel; photos depicting the injectable properties (d) and self-healing (e) properties of the hydrogel.

The rheological and viscoelastic behavior of PFP hydrogels was also investigated. During the frequency scanning process (Fig. 5a), PFP-0.33 exhibits an increase in storage modulus (G′) from approximately 720 Pa at 3.54 Hz to approximately 880 Pa at 44.9 Hz, before decreasing back to approximately 740 Pa at 100 Hz. In contrast, PFP-0.25 shows a gradual decline in G′ from approximately 484 Pa at 3.54 Hz to approximately 450 Pa at 38 Hz, followed by a rapid drop to nearly 0 Pa at 100 Hz. The observed decrease in modulus with increasing frequency indicates that the hydrogel structure behaves as a solid in the low-frequency range. However, during high-frequency vibrations, the polymer chains may not have sufficient time to reconfigure, resulting in a diminished resistance to deformation or stress within the hydrogel structure.

In all oscillation tests, including subsequent strain scans, it was observed that hydrogels with higher aldehyde group content exhibit a higher modulus. In the strain scanning (Fig. 5b), PFP-0.33 hydrogels demonstrate a slight weakening trend for both G′ and G″, which then rapidly increases with strain, indicating typical elastic hydrogel behavior. The PFP-0.25 hydrogel maintains a constant energy storage modulus of approximately 400 Pa (γ ≈ 0–70 %). Upon further deformation, the hydrogel exhibits strain-hardening behavior, with the energy storage modulus increasing to 420 Pa (γ ≈ 100–580 %), before rapidly decreasing to intersect with G″ (γ ≈ 670 %). This data indicates that while the network structure of the hydrogel remains intact, its fluidity is enhanced, allowing the hydrogel to transition between solid and fluid states, thereby facilitating injectability. Macroscopic experiments further confirm the injectable properties of the hydrogel (Fig. 5d). Moreover, in compression experiment testing, as illustrated in Fig. S7, the cylindrical PFP hydrogel exhibited no significant fracture when subjected to various loading pressures and was able to recover to its original shape.

The self-healing capacity of the PFP hydrogel was evaluated through a macroscopic experiment. Two disc-shaped PFP-0.25 hydrogels were each cut into three pieces. The freshly cut halves, differing in color, were placed in contact and positioned in a mold at 37 °C. After 3 h, the PFP-0.25 hydrogel had completely regenerated into a unified disc, visibly displaying color fusion (Fig. 5e).

To quantitatively assess the healing ability, rheological testing was conducted. Fig. S8 illustrates the G′ and corresponding healing efficiency (HE) values of PFP before and after healing at different time intervals at 37 °C. After a 1-hour healing period, the HE reached 50.8 %. Remarkably, after a 3-hour healing period, the G′ value was nearly equivalent to that of the original hydrogel, while the HE soared to 93.9 % (Fig. S8). To further evaluate the self-healing ability of the hydrogel, continuous stepped strain cycling was performed at a constant frequency of 1 Hz, using low (γ = 1 %) and high deformation (γ = 700 %) (Fig. 5c). At low deformation, the material exhibited typical elastic hydrogel behavior, with a G′ of 506 ± 5.5 Pa and a loss modulus (G″) of 167 ± 0.55 Pa. Upon applying 700% strain, the hydrogel broke instantaneously and displayed pronounced liquid-like behavior, with a G′ of 277 ± 2.3 Pa and a G″ of 328 ± 5.1 Pa. After 60 s of large deformation, the applied strain was reduced to 1 %. The storage modulus promptly increased to 523 ± 7.4 Pa, while the loss modulus decreased slightly (G″ ≈ 196 ± 3.5 Pa). This self-healing process is repeatable over several cycles, with the storage modulus fully restored after each hydrogel fracture. These findings exemplify the impressive self-healing capabilities of the hydrogel.

### 3.7 Cytocompatibility evaluation

**Fig. 6:**
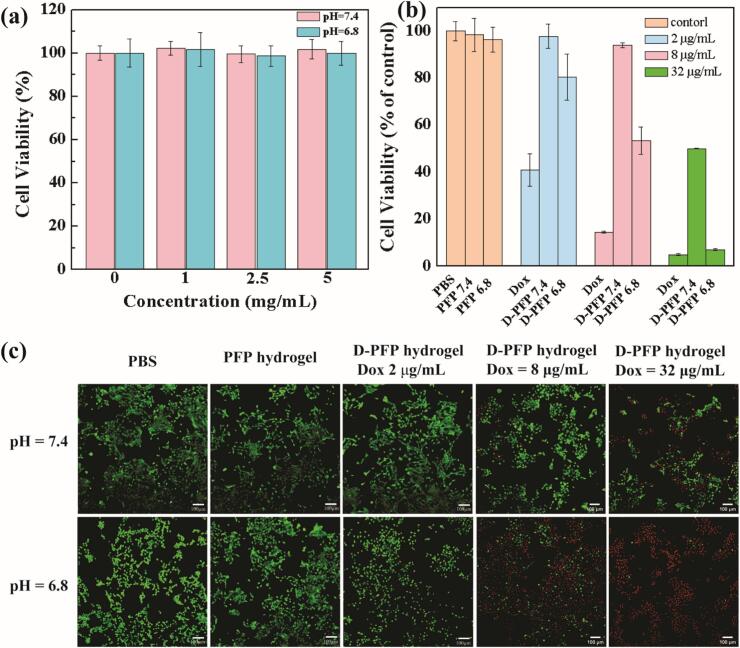
(a) Cytocompatibility assessment of PFP hydrogels with various extraction concentrations; (b) anti-cancer effect of D-PFP hydrogel; (c) In vitro calcein AM/PI co-staining with PBS, FPF hydrogel, D-PFP hydrogel at various Dox concentration.

The cytocompatibility of the hydrogel was assessed using the CCK-8 method. Fig. 6a illustrates the effect of various concentrations of hydrogel extract on the activity of L929 cells. At gel concentrations below 5 mg/mL both under the pH value of 7.4 and 6.8, no significant impact on cell activity was observed.

### 3.8 In vitro anti-cancer cells study

To examine the pH-dependent and anti-cancer properties of Dox released from hydrogels, C6 cells were utilized as a model in an assay employing a 48-hour gel extract obtained at various pH levels. The IC_50_ of Dox toward C6 cells was calculated to 2.04 μg/mL. The D-PFP hydrogel extract significantly influences cell activity, demonstrating a clear pH dependency. Specifically, at a Dox concentration of 2 μg/mL and 8 μg/mL Dox concentration, when exposed to the immersion solution at pH 6.8, the activity of C6 cells is notably lower than that was observed in the 7.4-gel group. Interestingly, at equivalent Dox concentrations, the cell activity of the 6.8-gel group surpasses that of the free Dox group, indicating an effective controlled release. In the high drug concentration group (32 μg/mL), pronounced pH-dependent drug release behavior was also noted. Moreover, although minor variations in cell activity were observed between the free Dox and pH 6.8 gel groups (4.93 % and 7.04 %, respectively), this difference did not translate into discernible variations in drug concentration. This outcome may be attributed to the elevated drug concentration exceeding the tolerance threshold of C6 cells.

The anti-cancer efficacy of the hydrogel was further investigated using live/dead co-staining assays (Fig. 6). As illustrated in the images, cells in the PBS and PFP hydrogel groups exhibited a substantial area of strong green fluorescence, indicating good biocompatibility. Conversely, for the D-PFP hydrogel, the area of strong green fluorescence diminished with increasing Dox concentrations at both pH 7.4 and pH 6.8. Notably, at Dox concentrations of 8 μg/mL and 32 μg/mL under pH 6.8, numerous cells were stained with red fluorescence, further demonstrating the hydrogel's pH-sensitive drug delivery properties.

## 4 Conclusions

Novel injectable self-healing hydrogels with enhanced stability and ultra-sensitive pH-dependent drug release were developed by mixing a benzaldehyde-terminated PVA-FBA solution with a PEI solution, resulting in the formation of dynamic benzoic-imine bonds. The optimal parameters for hydrogel preparation involved using PF-0.25 polymer, which has an aldehyde group content of 0.82 mmol/g, as the precursor. A 12 wt% precursor solution was crosslinked with a 5 wt% PEI solution. The inclusion of the conjugation effect of the benzene ring significantly reduces drug release under physiological conditions while demonstrating a markedly higher drug release rate at pH 6.8, thereby underscoring its superior pH sensitivity. Rheological assessments indicate that the hydrogel exhibits favorable injectability and self-healing properties. Cytocompatibility evaluations conducted on mouse fibroblast (L929) cells at a concentration of 5 mg/mL confirm the non-toxic nature of the hydrogel. In vitro drug release studies performed at varying pH levels establish the pH-dependent anti-cancer activity of the hydrogel. At pH 6.8, the hydrogel exhibits a significantly lower C6 cell activity than that was observed at pH 7.4. These findings highlight the considerable potential of these newly developed hydrogels for diverse biomedical applications. Their excellent stability, pH-responsive drug release, and controlled anti-cancer activity position them as promising candidates for future therapeutic interventions and targeted drug delivery systems.

## CRediT authorship contribution statement

**Yang Yu:** Writing – original draft, Formal analysis, Data curation. **Yili Zhao:** Writing – review & editing, Supervision, Funding acquisition, Formal analysis, Conceptualization. **Yujiao Zou:** Formal analysis, Data curation. **Chanyi Lu:** Writing – original draft, Formal analysis, Data curation. **Ni Li:** Writing – review & editing, Formal analysis, Conceptualization. **Zhiyuan Shi:** Writing – review & editing, Investigation, Funding acquisition. **Xin Li:** Writing – review & editing, Supervision, Funding acquisition, Formal analysis, Conceptualization. **Xixi Lai:** Writing – review & editing, Funding acquisition, Data curation, Conceptualization.

## Declaration of competing interest

The authors declare that they have no known competing financial interests or personal relationships that could have appeared to influence the work reported in this paper.

## Data Availability

Data will be made available on request.

## Appendix A Supplementary data

Supplementary material Image 1
