# Supplementary material for: Ultra-sensitive pH responsive hydrogels with injectable and self-healing performance for controlled drug delivery
Source: Int J Pharm X. 2025 Apr 15;9:100334. doi: 10.1016/j.ijpx.2025.100334 (PMC12416092; doi:10.1016/j.ijpx.2025.100334)
Supplement: Supplementary file 1 — Supplementary material [file mmc1.docx]

Supporting Information

**Ultra-sensitive pH responsive hydrogels with** **injectable and self-healing performance for controlled drug delivery**

*Yang Yu ^a,b,1^, Yili Zhao ^b,1^*, *Yujiao* *Zou ^b^, Chanyi* *Lu ^b^, Ni Li ^b^, Zhiyuan Shi ^c^*, Xin Li ^d^*, and Xixi Lai ^a^**

^a^ Department of Respiratory and Critical Care Medicine, The First Affiliated Hospital of Wenzhou Medical University, Wenzhou 325015, China

^b^ State Key Laboratory of Bio-based Fiber Materials, College of Textile Science and Engineering, Zhejiang Sci-Tech University, Hangzhou 310018, China

^c^ Tianjin Key Laboratory of Drug Delivery & High-Efficiency, School of Pharmaceutical Science and Technology, Tianjin University, Tianjin 300072, China

^c^ Department of Mechanical and Automation Engineering, The Chinese University of Hong Kong, Hong Kong 999077, China

1 These authors contributed equally to this work.

* To whom correspondence should be addressed. Email: zhiyuan_shi2023@tju.edu.cn (Z. Shi), xli@dwi.rwth-aachen.de (X. Li), laiqianqian@wzhospital.cn (X. Lai).

## Characterization techniques

UV-vis spectrometry (Lambda 950 UV-vis spectrometer) was performed to characterize the concentration of the Dox in the immersing solution *via* the measurement of the absorbance. The intensity of each concentration (5-100 μg/ml) is measured at 482 nm to establish a standard curve.

Fourier Transform Infrared (FTIR) spectroscopy was carried out using a Thermo Nicolet FTIR (NICOLET Is50) to investigate the binding capacity of PF samples with Dox. To evaluate the binding capacity of PF samples with Dox, 30 mg of DOX, having undergone prior desalination, was introduced into 5 mL PF solution (PF-0.25, PF-0.33, 12wt%). The mixture underwent agitation within a light-deprived setting at 37 ^o^C for 20 minutes. Subsequently, the resultant mixture was transferred to a drying oven, set at 40^o^C, where it was subjected to sustained desiccation until the formation of a film ensued. 0.5 g of the composite membrane was transferred into a dialysis bag (MWCO 8000-14000). This operation was concomitant with the addition of 5 mL of deionized water, in preparation for the ensuing phases of experimentation. The investigation into the interaction between DOX and PF was conducted by its drug release rate. Submerging the dialysis bag into a PBS solution (pH 7.4, 100mL), and maintained at 37^o^C. Periodically, an approximate 5% aliquot of the dialysate volume was extracted from the PBS solution, followed by replenishment of the total volume through the addition of fresh PBS. In tandem, an equivalent control experiment was conducted, employing a 5 mL PVA solution (12wt%), which strictly adhered to the identical procedural regimen.


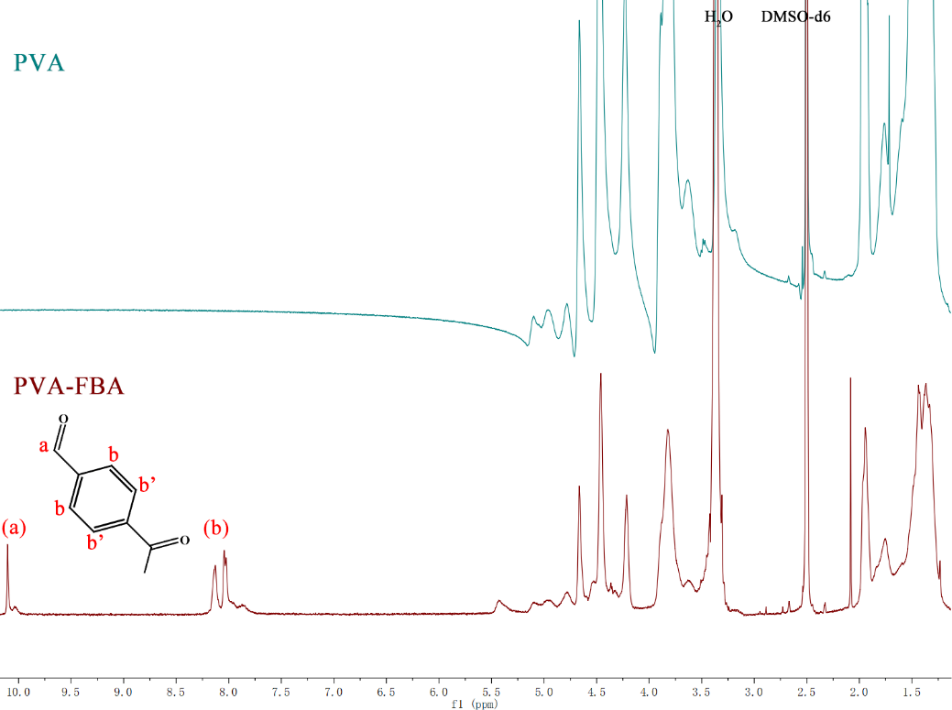


Figure S1. ^1^H NMR spectrum of the PVA and PVA-FBA.


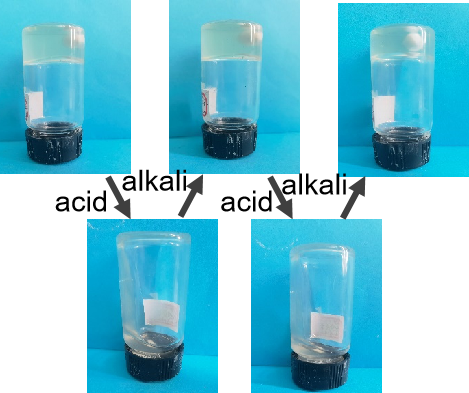


Figure S2. The sol-gel transformation of PFP hydrogels occurred with the change of pH.


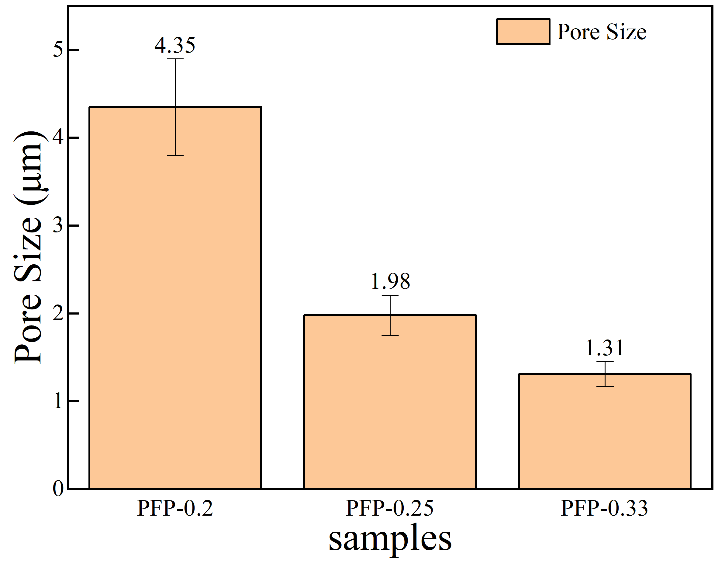


Figure S3. The pore size histogram of PFP hydrogels.


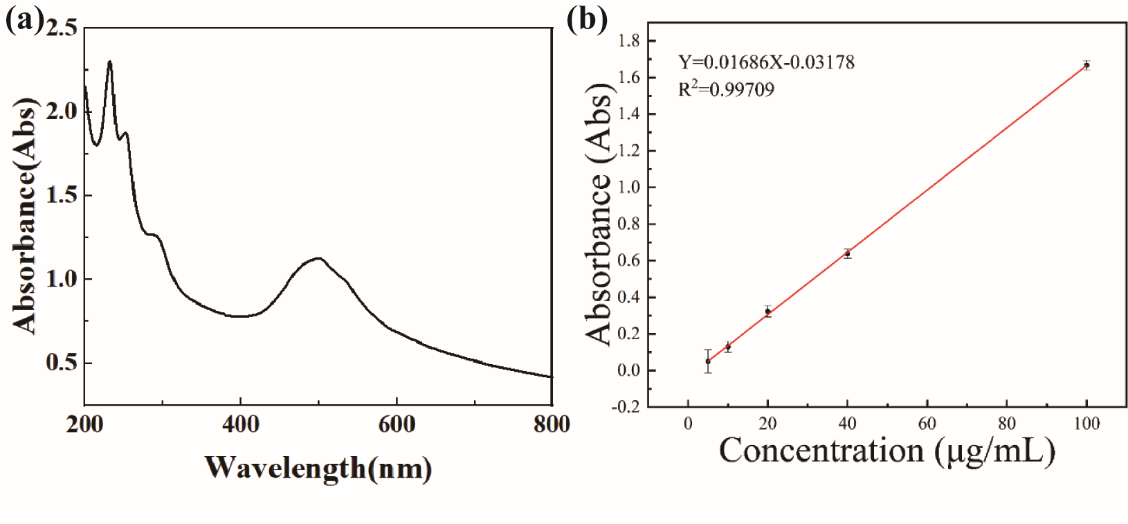


Figure S4. UV-vis spectra of Dox and absorbance-concentration standard curve.


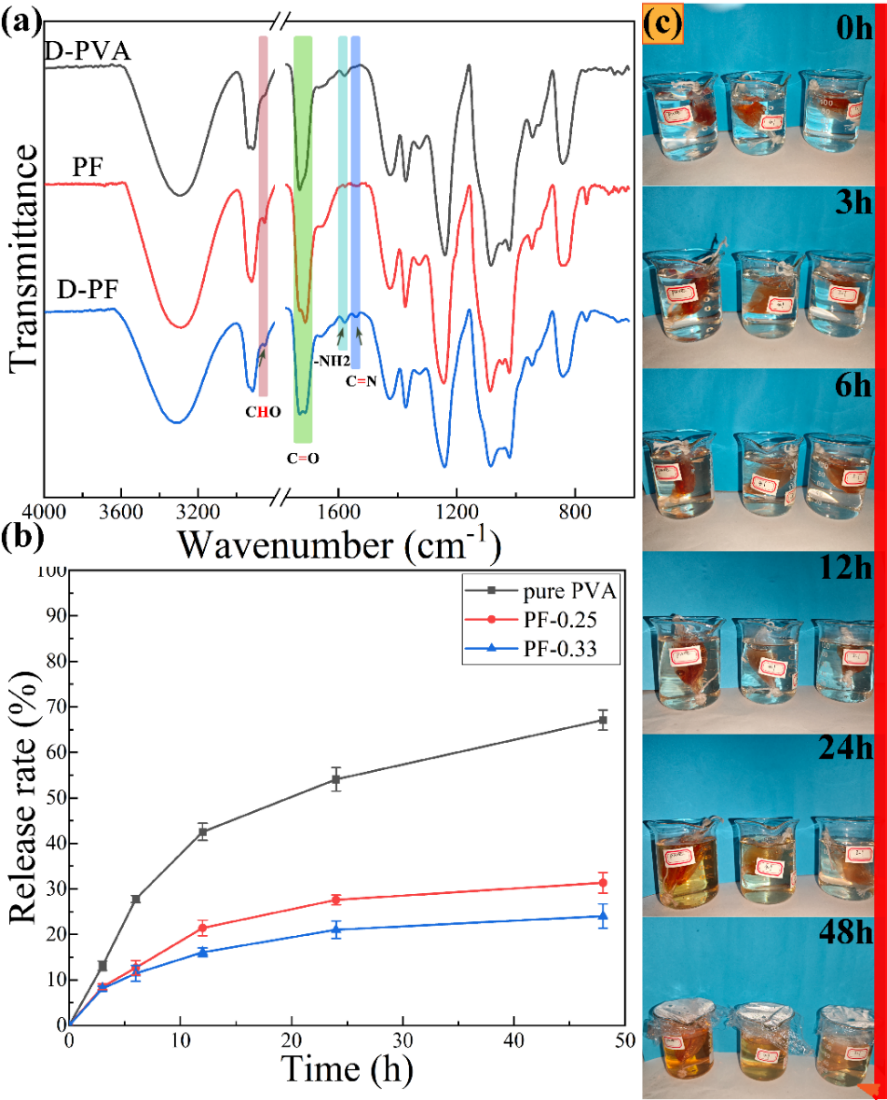


Figure S5. (a) FTIR comparative analysis of D-PVA, PF and D-PF samples; (b) 48-hour drug release curve of PVA, PF-0.25, and PF-0.33 specimens mixed with DOX; (c) digital photograph of PVA (left), PF-0.25 (middle), and PF-0.33 (right) specimens mixed with DOX.


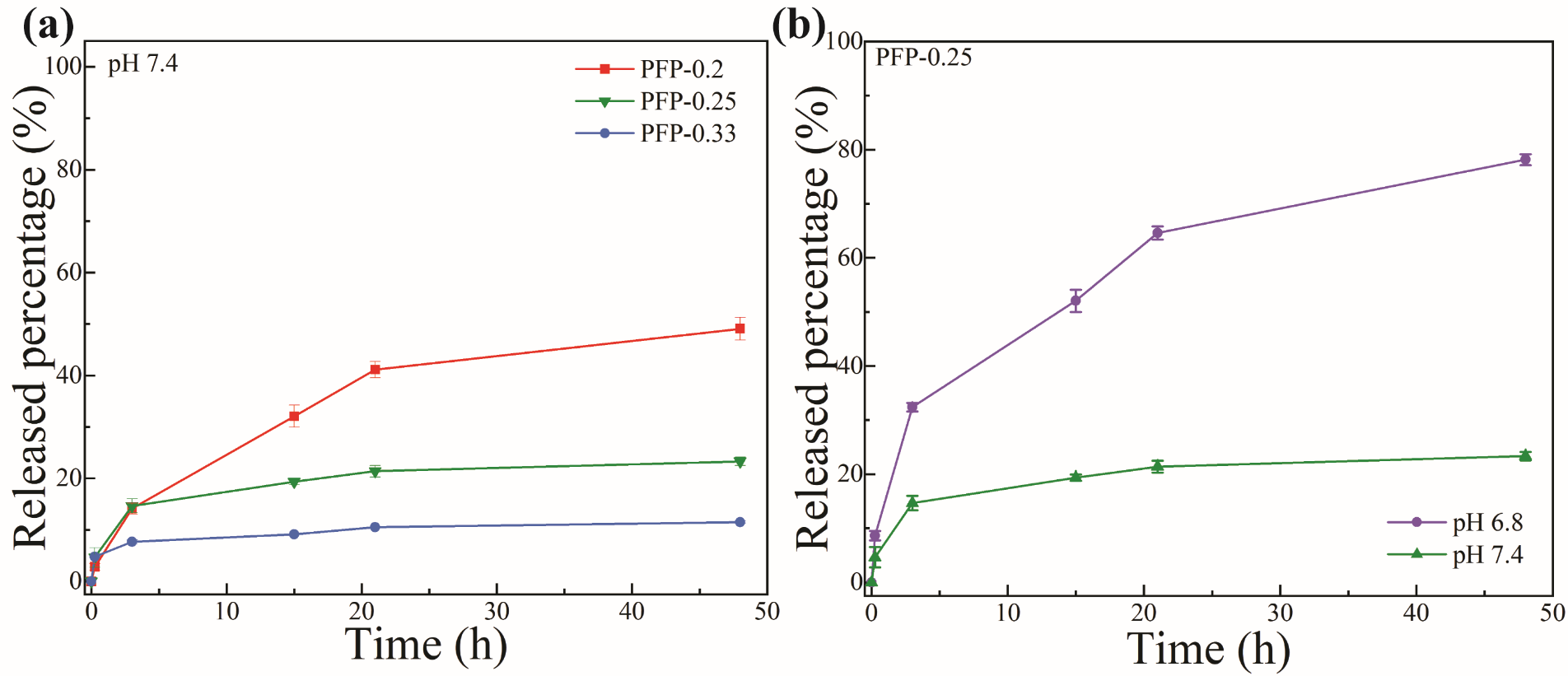


Figure S6. (a) Drug release percentage of different PFP hydrogels at pH 7.4; (b) drug release rate of PFP-0.25 at different pH levels.


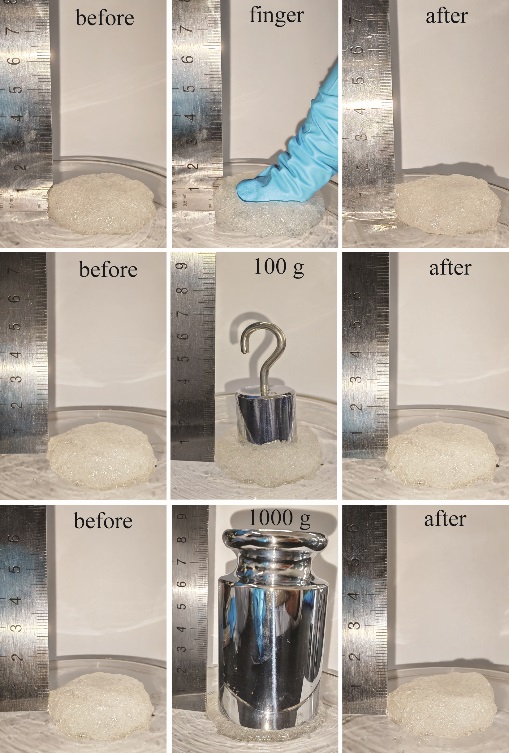


Figure S7. Visual compression testing of PFP hydrogel under different loading pressure.


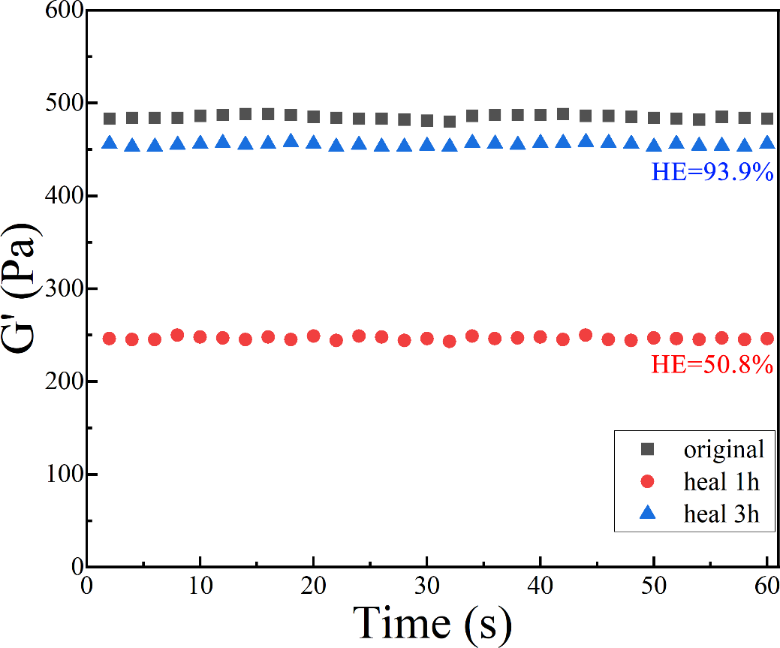


Figure S8. G’ and HE of PFP hydrogel before and after healing.
